# Hippocampal Chandelier Cells Modulate Seizure Susceptibility and Severity

**DOI:** 10.1002/advs.202501066

**Published:** 2025-10-29

**Authors:** Yang Li, Jifeng Tian, Jiafan Wei, Qianyun Wang, Jiaxu Ge, Jianguang Ni, Jiangteng Lu, Yilin Tai

**Affiliations:** ^1^ State Key Laboratory of Brain Function and Disorders and MOE Frontiers Center for Brain Science and the Institutes of Brain Science Fudan University Shanghai 200032 China; ^2^ Department of Pharmacology and Chemical Biology Shanghai Jiao Tong University School of Medicine Shanghai 200025 China; ^3^ Songjiang Hospital and Songjiang Research Institute Shanghai Key Laboratory of Emotions and Affective Disorders Shanghai Jiao Tong University School of Medicine Shanghai 201600 China; ^4^ Department of Anatomy and Physiology Shanghai Jiao Tong University School of Medicine Shanghai 200025 China; ^5^ Department of Neurosurgery Huashan Hospital Fudan University Shanghai 200032 China

**Keywords:** axon initial segment, chandelier cells, seizure

## Abstract

The axon initial segment (AIS), serving as the site for initiating action potentials (APs), is a key target for efficient regulation of neuronal activity. In mammals, chandelier cells (ChCs) represent a distinct subtype of GABAergic interneurons that selectively target the AIS of projection neurons (PNs). This strategic property endows them with the potential to effectively control the firing of PNs. They respond to diverse physiological stimuli and undergo homeostatic plasticity during network hyperactivity. However, their response in pathological states, such as epileptic seizures, remains unclear. In this study, we observed an increase in the ChC Ca^2+^ signal following the rise of ictal discharges. Blocking ChC synaptic transmission in the hippocampal CA1 region placed animals in a subthreshold status to develop seizures and heightens susceptibility and severity to kainic acid (KA)‐induced epilepsy. Furthermore, bidirectional chemogenetic modulation of ChCs altered seizure susceptibility, supporting the hypothesis that ChCs safeguard the network activity. Notably, boosting ChC activity during the chronic phase mitigated spontaneous seizures. Additionally, intensified ChC‐AIS innervation is observed following status epilepticus (SE), suggesting a homeostatic protective role of ChCs. The findings revealed an active anti‐ictogenic role of ChCs during seizures, highlighting their protective function in pathological conditions.

## Graphical abstract

The axon initial segment (AIS), crucial for action potential initiation, serves as a prime locus for controlling network activity. Chandelier Cells (ChCs), evolved in the mammals, intricately target AISs, acting as gatekeepers for neuronal firing. Diminishing ChC input makes animals more prone to developing seizures, whereas fortifying this connection offers long‐lasting protective benefits against seizures.

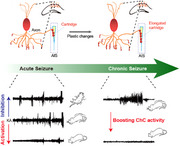

## 1 Introduction

The foundation of neuronal communication lies in the firing of action potentials (APs), which are preferentially initiated at the AIS, the proximal domain of the axon.^[^ ^1^, ^2^, ^3^, ^4^, ^5^ ^]^ Serving as a command center, direct regulation of the AIS has a profound impact on network activity.^[^ ^6^, ^7^, ^8^, ^9^, ^10^, ^11^, ^12^, ^13^, ^14^ ^]^ In mammals, a distinctive subtype of GABAergic interneuron, known as chandelier cells, has evolved to selectively target the AIS of the PNs, adding a new dimension to the regulation of AP firing.^[^ ^15^, ^16^ ^]^ A single ChC is capable of innervating hundreds of PNs in the cortex and up to 1200 PNs in the hippocampus, endowing it with remarkable potential as an efficient modulator of network activity.^[^ ^16^, ^17^, ^18^, ^19^ ^]^

Multiple lines of evidence, at both structural and functional levels, suggest that ChCs may serve as surveillance of network activity and prevent excessive excitatory activity. In vivo recordings show that ChCs fire more robustly than other neuron types when network excitation increases^[^ ^20^ ^]^ and activation of ChCs in vivo primarily leads to strong inhibition of nearby PNs.^[^ ^21^, ^22^, ^23^ ^]^ Additionally, ChCs undergo homeostatic plasticity to strengthen their inhibitory connections when PNs are persistently activated.^[^ ^24^ ^]^ Notably, alterations in ChC activity trigger homeostatic plasticity at the AIS as well.^[^ ^25^ ^]^ These findings suggest that ChCs may actively contribute to maintaining balanced network activity under normal physiological conditions. However, whether they sustain this regulatory role in pathological states, such as during epileptic seizures, remains unknown.

Decades ago, it was proposed that altered ChCs connections might contribute to seizure onset, giving rise to the “chandelier cell hypothesis” of epilepsy.^[^ ^26^ ^]^ Indeed, studies have shown the loss of ChCs or rearrangement of ChC cartridges in patients with epileptic seizures.^[^ ^27^, ^28^, ^29^, ^30^ ^]^ Mice with specific deletion of ErbB4, a key regulator of ChC synaptic formation, in parvalbumin (PV) positive cells, which include both ChCs and PV^+^ basket cells, are more susceptible to developing seizures.^[^ ^31^, ^32^ ^]^ Recent observations in animal models of temporal lobe epilepsy (TLE) have shown compromised ChC input and output activities in the dentate gyrus (DG) following SE.^[^ ^33^ ^]^ However, it remains unclear whether the changes of ChCs represent crucial factors that increase the susceptibility to seizure onset or merely a consequence of epileptic seizures.

In this study, we employed a recently developed genetic strategy to efficiently label and manipulate ChCs.^[^ ^34^, ^35^ ^]^ Our findings revealed that the activity of ChCs increased following KA‐induced seizures, indicating ChCs were recruited during ictal episodes, mirroring their response to synchronized PN activity in physiological conditions. Interfering with ChC to AIS synaptic transmission through expression of tetanus toxin (Tettox) led to the occurrence of spontaneous recurrent seizures (SRSs), affirming the essential role of ChCs in maintaining a balanced network activity. In the KA‐induced TLE model, impaired ChC to AIS transmission increased the susceptibility to seizure development. Conversely, enhancing ChC activity reduced seizure severity. During the chronic phase of epilepsy, the protective role of ChCs persisted, as increasing ChC activity resulted in reduced incidence of spontaneous seizures. These findings unveil the protective function of ChCs in pathological conditions and propose a novel approach for alleviating seizures through boosting ChC activity.

## 2 Results

### 2.1 ChCs were Recruited Following Seizure Onset

**Figure 1:**
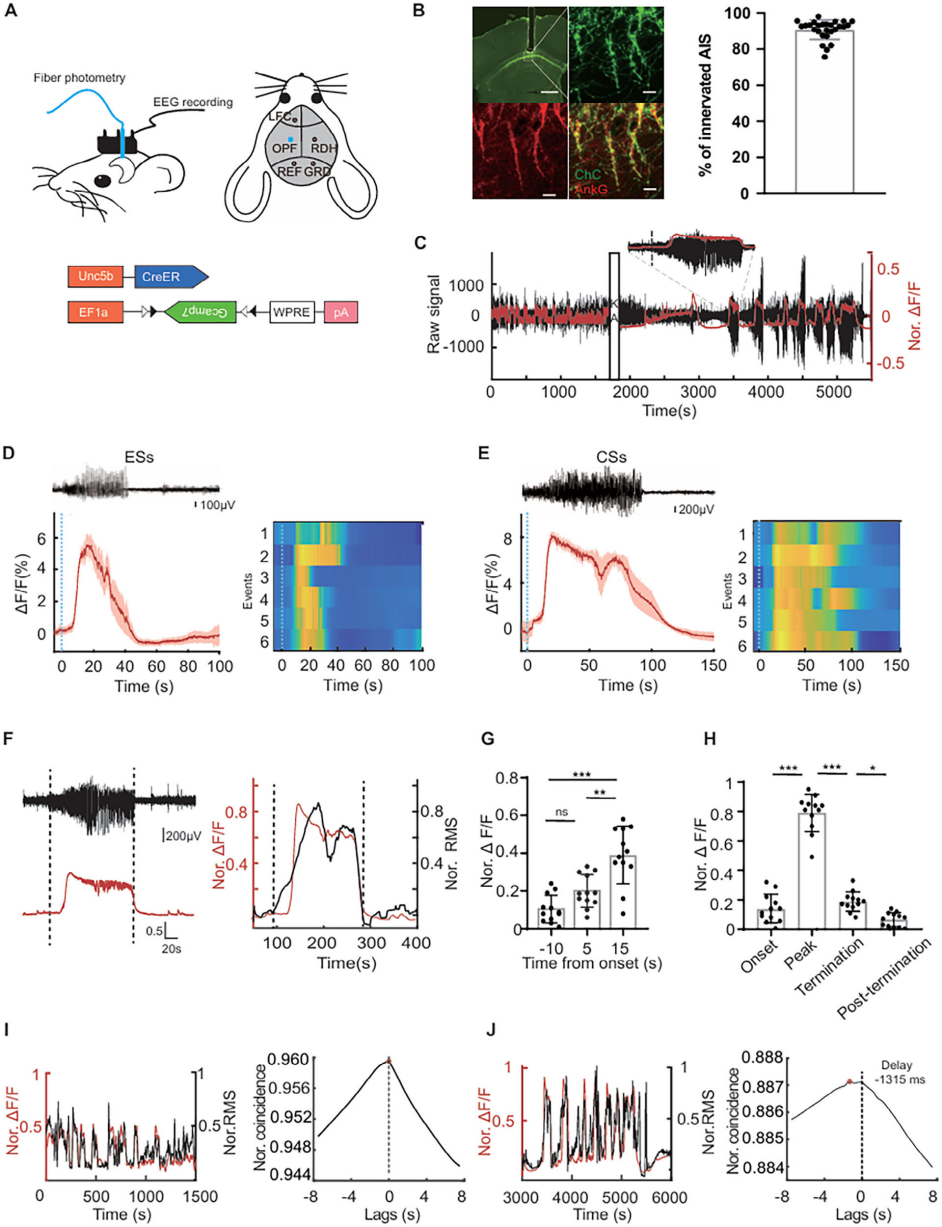
Recruitment of ChCs during epileptic seizures. A) Schematic representation of simultaneous fiber photometry and EEG recordings performed in *Unc5b‐CreER* mice infected with AAV virus encoding the Cre dependent DIO‐Gcamp7. The left panel illustrates the experimental setup in mice, while the right panel shows the placement of subdural screw electrodes and an optical fiber (OPF) for fluorescence collection. (GRD: ground, LFC: left frontal cortex, RDH: right dorsal hippocampus, REF: reference). B) Representative images illustrating the injection site of AAV‐DIO‐Gcamp7 in the CA1 region (scale bar: 500 µm) and the percentage of AISs been innervated by viral infected ChCs. Infected ChCs were featured by their axonal terminals (green) specifically targeting AIS with AnkG staining (red, scale bar: 5 µm). C) Representative traces of EEG (black) and Ca^2+^ signals (red) in mice before and after kainic acid (KA) injection. The inset depicts a magnified time scale illustrating the onset of an ictal event (dashed line). D) Representative EEG (upper) and average Ca^2+^ signals (lower) during ESs. The dashed line indicates seizure onset. Heatmap on the right panel showed Ca^2+^ signals of each ES incident (n = 6, from 3 mice). E) Representative EEG (upper) and average Ca^2+^ signals (lower) during CSs. The dashed line indicates seizure onset. Heatmap on the right panel showed Ca^2+^ signals of each CS incident (n=6, from 3 mice). F) Left: Ca^2+^ signals increased following seizure onset during an ictal event. Right: Normalized Ca^2+^ signals and amplitude RMS depiction. The dashed line indicates seizure onset and termination, respectively. G) Ca^2+^ signals at 20 s following seizure onset showed a significant increase (one way ANOVA, ‐5 sec versus 10 sec, *P* = 0.3901; ‐5 sec versus 15 sec, *P* = 0.0279; 10 sec versus 15 sec, *P* = 0.0044, n = 12, from 3 mice). H) Change of Ca^2+^ signals at seizure onset, peak, termination and post‐termination (one way ANOVA, onset versus peak and peak versus termination *P* <0.0001, termination versus post‐termination, *P* = 0.0991, n = 12, from 3 mice). I,J) Changes in EEG and Ca^2+^ signals before and after KA injection. The cross correlation and delay value between RMS power and Ca^2+^ signals during the freely moving state before KA injection (I) and during seizures after KA injection (J). Data are represented as mean ± SD. ns *P* > 0.05, *P < 0.05, ** *P* < 0.01, *** *P* < 0.001.

To investigate how ChCs respond to ictal events, we combined EEG recordings with fiber photometry recordings of bulk Ca^2+^ imaging to track the activity pattern of ChCs in the hippocampal CA1 region during seizures (**Figure** **1**A). We used *Unc5b‐CreER* driver line, in conjugation with an adeno‐associated virus serotype 9 (AAV9) encoding Cre‐dependent Gcamp7s (AAV‐DIO‐Gcamp7s) to selectively express the Ca^2+^ indicator in ChCs (Figure 1A,B), as detailed in recent reports.^[^ ^22^, ^35^ ^]^ Epileptic seizures were induced using the classic KA‐induced TLE model, where KA (25 mg kg^−1^) was administered via intraperitoneal (i.p.) injection. ^[^ ^36^, ^37^ ^]^ Seizures appeared within 40 min following KA administration. The activity pattern of ChCs during seizures was depicted by concurrently capturing EEG signals (electrodes implanted above the left frontal cortex and right dorsal hippocampus) and Ca^2+^ signals from the CA1 ChCs (Figure 1C).

Large Ca^2+^ signals were detected in ChCs during both electrographic seizures (ESs) and convulsive seizures (CSs), with the CSs exhibiting more pronounced and prolonged activity (Figure 1D,E). Through aligning and averaging the EEG and photometry recordings, we observed a delayed buildup of Ca^2+^ signals in ChCs following the rise of the root mean square (RMS) of EEG signals (Figure 1F; Figure S1A–C, Supporting Information). This phenomenon was different from that observed in the PV^+^ population, which exhibited early Ca^2+^ buildup even before the rise of RMS (Figure S1D–H). Analysis of RMS values indicated minimum fluctuation in ChC Ca^2+^ signals 5 s before and 10 s after seizure onset, followed by a rise in Ca^2+^ signals starting ≈15 s after the onset of the seizure (Figure 1G; Figure S1A, Supporting Information). The ChC Ca^2+^ signals at 15 s post seizure onset showed a significant increase compared to those at 10 s post onset (Figure 1G). A marked surge in Ca^2+^ signals was evident at the peak of the seizure (Figure 1H).

Notably, both before and after KA administration, the amplitude of the Ca^2+^ signals exhibited a positive correlation with the RMS of the electrical discharges (Figure 1I,J). Cross‐correlation analysis showed that the delay value (RMS power leads Ca^2+^ signal) at which the correlation was maximal is near zero before KA administration, whereas it shifted to −1215 ms on average afterward KA administration (Figure 1I,J; Figure S2, Supporting Information). This finding suggests a differential association between ChC activity and electrographic power in healthy states and pathological seizures.

### 2.2 Reducing ChC Synaptic Transmission by Tettox Heightened Susceptibility to Seizures

**Figure 2:**
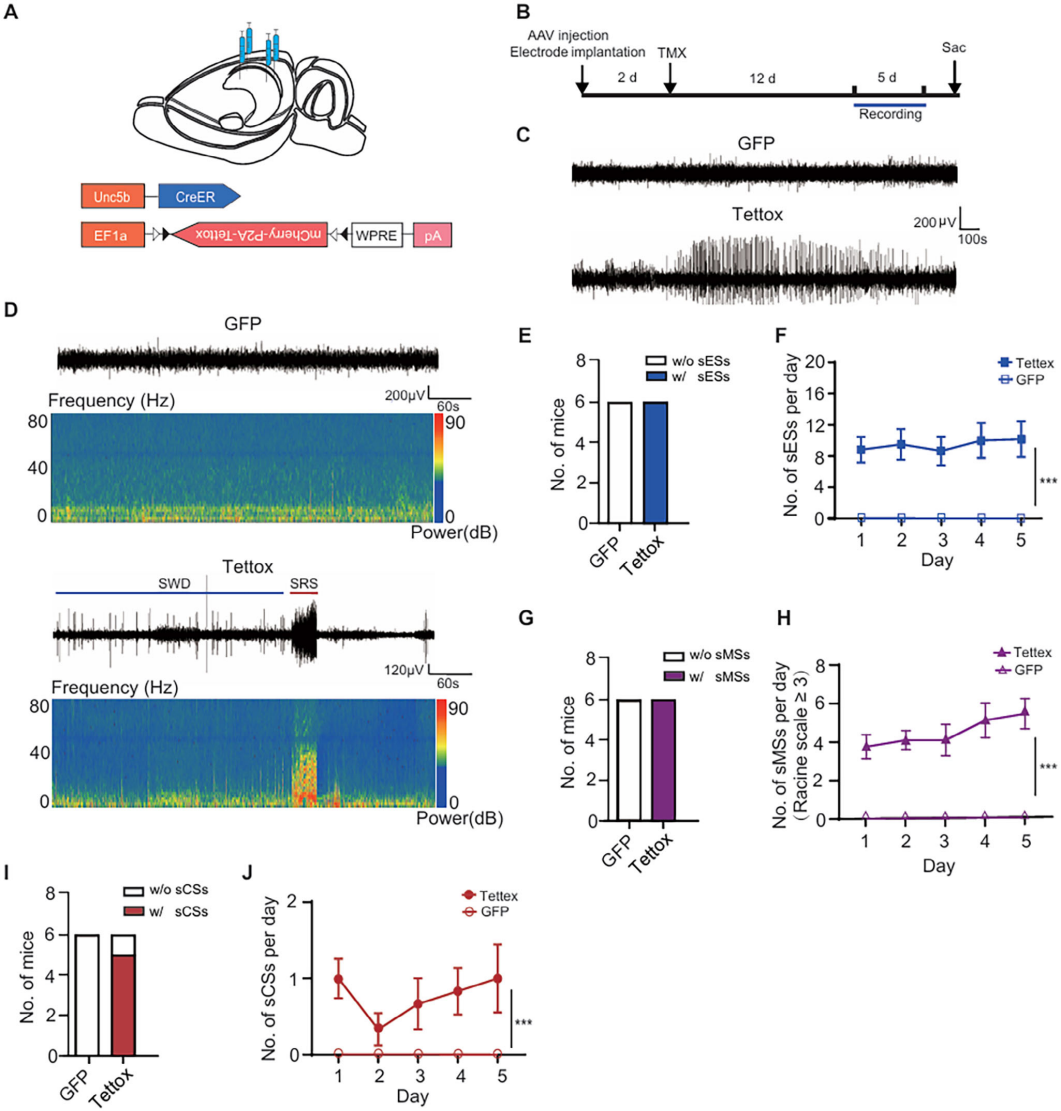
Blocking ChC synaptic transmission with Tettox led to spontaneous seizures. A) Schematics illustrating the sites of AAV‐DIO‐Tettox injection and its expression system. B) Experimental setup illustrating the timeline of electrode implantation along with viral injection, and EEG recording performed for 5 consecutive days. C) Representative EEG recordings from mice infected with AAV encoding DIO‐GFP or DIO‐Tettox. The lower panel shows the emergence of an SWD in the Tettox group. D) Representative EEG traces and the corresponding spectrograms from mice infected with AAV encoding DIO‐GFP or DIO‐Tettox, an SRS following the SWD was depicted in the Tettox group. E) Number of mice exhibiting sESs in GFP control group and Tettox group (GFP: n = 6; Tettox: n = 6). F) Occurrence of sESs per day in the Tettox group (Two‐way ANOVA, *P* < 0.001, GFP: n = 6; Tettox: n = 6). G) Number of mice exhibiting sMSs (Racine scale ≥ 3) in GFP control group and Tettox group (GFP: n = 6; Tettox: n = 6). H) Occurrence of sMSs (Racine scale ≥ 3) per day in the Tettox group (Two‐way ANOVA, *P* < 0.001, GFP: n = 6; Tettox: n = 6). I) Number of mice exhibiting sCSs in GFP control group and Tettox group (GFP: n = 6; Tettox: n = 6). J) Occurrence of sCSs per day in the Tettox group (Two‐way ANOVA, *P* < 0.001, GFP: n = 6; Tettox: n = 6). Data are represented as mean ± SD. ^***^ *P* < 0.001.

To gain insight into whether ChCs serve as a safeguard of the network activity, we first explored the consequence of reducing ChC synaptic transmission. We bilaterally injected four sites of the CA1 region with AAV encoding a Cre‐dependent tetanus toxin (DIO‐Tettox‐P2A‐mCherry) in the *Unc5b‐CreER* mice (**Figure** **2**A). The expression of Tettox was confined to ChCs and effectively cleaved VAMP2, a key component of the SNARE complex essential for presynaptic vesicle release, within a two‐week timeframe (Figure S3, Supporting Information). Then, EEG signals were recorded for 5 consecutive days to explore the instant effect of reducing ChC synaptic transmission (Figure 2B). Spike and wave discharges (SWDs), characterized by a rhythmic pattern repeating at a frequency above 2 Hz, where each cycle comprises a high‐voltage spike followed by a slower wave, were observed during recordings in the group infected with AAV‐DIO‐Tettox, but were absent in the control group (Figure 2C). Electrographic seizures (ESs) are identified when the RMS amplitude exceeded three standard deviations (3×SD) above baseline between 10–40 Hz for more than 5 s. Spontaneous recurrent seizures (SRSs), characterized by unprovoked, repetitive spontaneous seizure episodes that occur in the absence of acute precipitating factors, are a hallmark of chronic epilepsy., It has been reported that SRSs can occur subsequent to some of the SWDs ^[^ ^38^ ^]^ as observed in our experimental conditions (Figure 2D). All animals in the Tettox group experienced spontaneous electrographic seizures (sESs), with an average occurrence of 9.434 times per day (Figure 2E,F). Video recordings were performed simultaneously with EEG recordings of mice individually housed in a recording chamber. These recordings revealed that some of the sESs detected by EEG were accompanied by spontaneous motor seizures (sMSs). Specifically, limbic motor seizures with rhythmic convulsions of the body correspond to Racine scale stage 3, while rearing with tonic‐clonic convulsions corresponds to Racine scale stage 4, and prolonged seizures culminating in SE correspond to Racine scale stage 5. Stages 4‐5 are also categorized as convulsive seizures (CSs). All animals in the Tettox group displayed sMSs at the Racine scale level of 3 or above, with an average occurrence of 4.56 times per day (Figure 2G,H). Among these animals, five out of six animals exhibited spontaneous convulsive seizures (sCSs), with an average occurrence of 0.766 times per day (Figure 2I,J).

**Figure 3:**
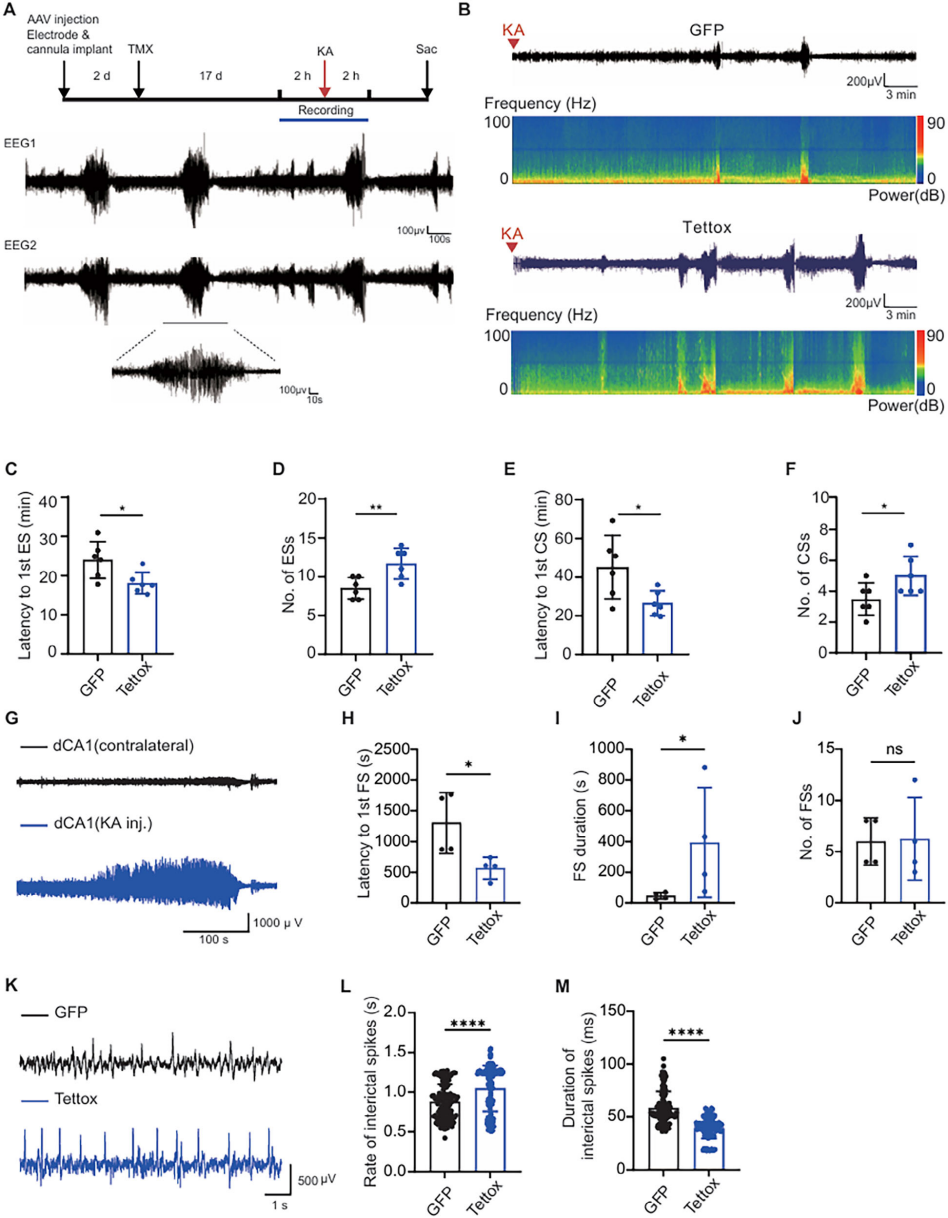
Blocking ChC synaptic transmission increased KA‐induced seizure susceptibility. A) Experimental setup illustrating the timeline of electrode and cannula implantation along with viral injection, and EEG recording performed before and after KA administration. B) Representative EEG traces and corresponding spectrograms of the GFP control group and Tettox group. C) Reduced latency to the first ES in the Tettox group following KA administration (Student's *t*‐test, *P* = 0.0465, GFP: n = 6; Tettox: n = 6). D) Increased frequency of ESs in the Tettox group following KA administration (Student's *t*‐test, *P* = 0.0090, GFP: n = 6; Tettox: n = 6). E) Latency to the first CS was shortened in the Tettox group (Student's *t*‐test, *P* = 0.0260, GFP: n = 6; Tettox: n = 6). F) Number of CSs was increased in the Tettox group (Student's *t*‐test, P = 0.0493, GFP: n = 6; Tettox: n = 6). G) Representative LFP traces showing an event of FS featured by asynchronous epileptiform activity in the left and right dCA1. H) Latency to first focal seizure onset (Mann‐Whitney U test, *P* = 0.0286, GFP: n = 4; Tettox: n = 4). I,J) Duration (I) and number (J) of FS events (Mann‐Whitney U test, I: *P* = 0.0286; J: *P* = 0.8286, GFP: n = 4; Tettox: n = 4). K) Representative traces of interictal spiking in GFP and Tettox group. L,M) Rate of interictal spiking (L) and duration (M) of each interictal spiking (Mann‐Whitney U test, *P* < 0.0001, GFP: n = 4 mice, 123 channels; Tettox: n = 4 mice, 125 channels; channel‐based statistics). Data are represented as mean ± SEM. ns *P* > 0.05, ^*^ *P* < 0.05, ^**^ *P* < 0.01, ^****^ *P* < 0.0001.

Next, we investigated whether the mice with reduced ChC synaptic transmission were more prone to develop seizures upon intrahippocampal KA induction, a widely used mouse model of TLE known for its reliability in triggering seizure onset. Administration of KA (100 ng, 0.2 µL) through a cannula to the CA1 region was applied to induce SE (**Figure** **3**A). Seizures were successfully induced in both the control group and Tettox group. A shorter latency to the first ES was found in mice infected with Tettox compared to the mice in the control group (Figure 3B,C). Moreover, they exhibited a higher frequency of ESs during the 2 h recorded period (Figure 3D). CSs are defined if video recordings depicted behaviors that are consistent with stages 4‐5 on the Racine scale. Consistently, the Tettox group had a shorter latency to develop CSs and experienced more of them (Figure 3E,F). Furthermore, the reduction in ChC synaptic transmission also affected focal seizures (FSs) as detected by local field potential (LFP) recordings (Figure 3G). In the Tettox group, the latency to the first FS was shortened (Figure 3H). While the frequency of FSs remained unchanged, the duration of the FS episodes was longer (Figure 3I,J). Notably, more interictal spiking with shorter duration was observed in the Tettox group compared to the control group (Figure 3K–M). These results suggest that reducing ChC synaptic transmission increases seizure susceptibility and severity.

### 2.3 Altered ChC Activity Affected Seizure Susceptibility and Severity

**Figure 4:**
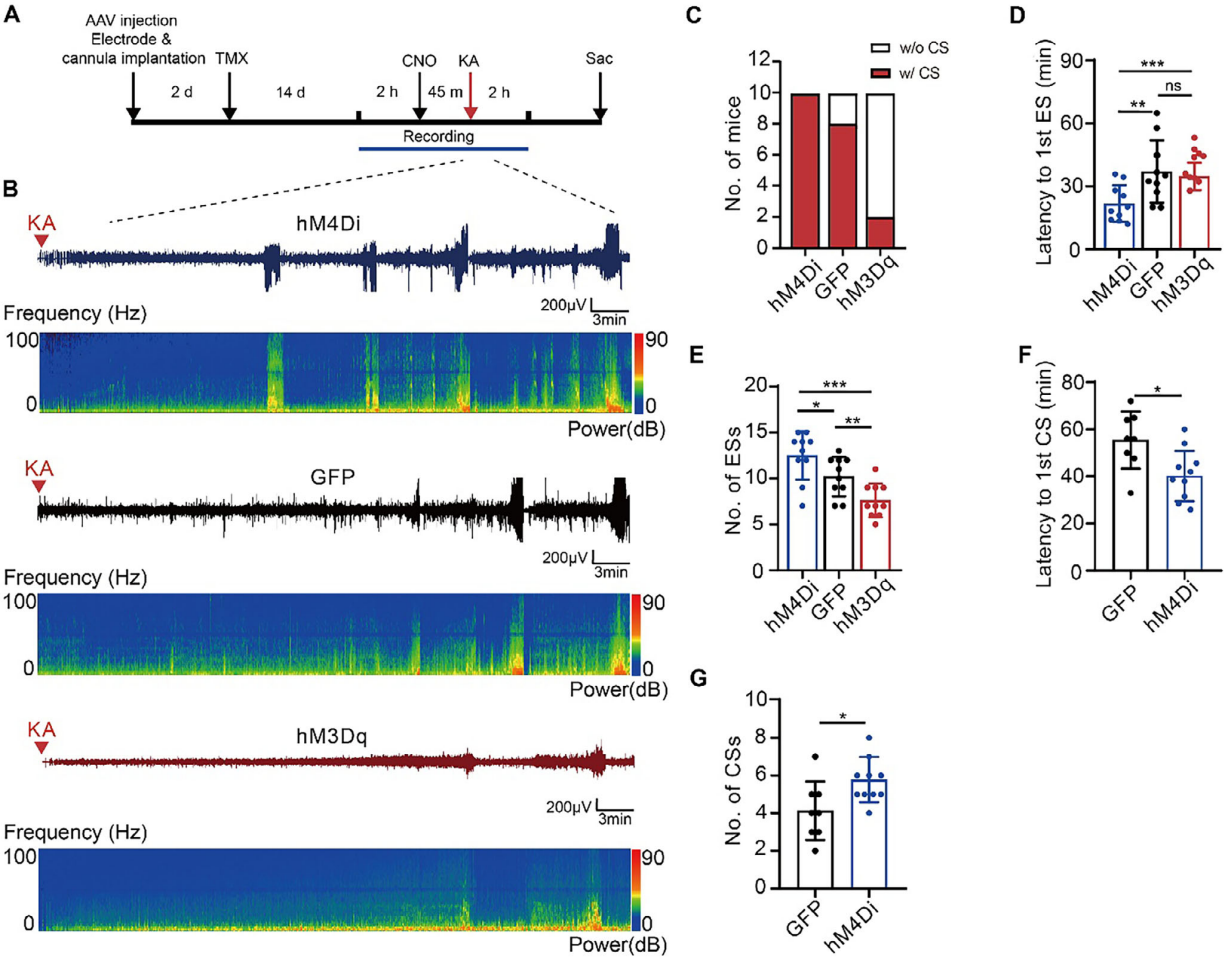
Effects of chemogenetic modulations of ChCs on acute seizures following KA administration. A) Experimental setup illustrating the timeline of electrode and cannula implantation along with viral injection, and EEG recording performed before and after KA administration. B) Representative EEG traces and corresponding spectrograms following application of CNO in the hM4Di, GFP or hM3Dq group. C) Statistics showing the number of mice experiencing CSs in each group (hM4Di: n = 10; GFP: n = 10; hM3Dq: n = 10). D) Latency to the first ES in each group (hM4Di versus GFP, *P* = 0.0095; hM4Di versus hM3Dq, *P* = 0.0297; GFP versus hM3Dq, *P* = 0.8784, one‐way ANOVA). E) Frequency of ESs in each group (hM4Di versus GFP, *P* = 0.0464; hM4Di versus hM3Dq, *P* = 0.0001; GFP versus hM3Dq, *P* = 0.038, one‐way ANOVA). F) Latency to the first CS in indicated group (Student's *t*‐test, *P* = 0.0119). G) Frequency of CSs in indicated group ((Student's *t*‐test, *P* = 0.0253.). Data are represented as mean ± SD. ^*^ *P* < 0.05, ^**^ *P* < 0.01, ^***^ *P* < 0.001.

Given that activation of ChCs can greatly reduce the firing rate of PNs in the CA1 region.^[^ ^23^ ^]^ We next investigate whether changing ChC excitability through chemogenetic approaches would result in differences in seizure susceptibility and severity. We employed a bidirectional chemogenetic approach using DREADDs (designer receptors exclusively activated by designer drugs, DREADDS).^[^ ^39^, ^40^ ^]^ The expression of hM4Di or hM3Dq was predominantly restricted to ChCs rather than Pv^+^Satb1^+^ basket cells or SOM^+^ interneurons (Figure S4, Supporting Information). ChCs infected with DIO‐hM4Di exhibited reduced excitability, whereas those infected with DIO‐hM3Dq showed increased excitability following CNO administration. These changes were confirmed by whole‐cell patch recordings of virally infected ChCs in hippocampal slices (Figure S5, Supporting Information). CNO was administered 45 min before KA administration through the intrahippocampal cannula in animals infected with DIO‐GFP, DIO‐hM4Di or DIO‐hM3Dq (**Figure** **4**A). All the mice in three different groups underwent ESs (Figure 4B). However, when considering CSs, only 20% of the mice with increased ChC activity (hM3Dq group) developed CSs, whereas 80% of the control mice did. In contrast, all mice with decreased ChC activity (hM4Di group) exhibited CSs (Figure 4C).

Similar to blocking ChC synaptic transmission, inhibition of ChCs significantly increased seizure susceptibility, as evidenced by shorter latency to the first ES and CS, along with a higher incidence of ESs and CSs (Figure 4D–G). These findings suggest that inhibition of ChCs exacerbates epilepsy susceptibility and severity. Conversely, although there was no significant difference in the latency to the first ES between the hM3Dq group and the control group (Figure 4D), notably fewer mice in the hM3Dq group experienced CSs, and the frequency of ESs was significantly lower compared to the control group (Figure 4C,E). Since only a limited number of animals in the hM3Dq group developed CSs, the latency and the frequency of CS were not further characterized.

In summary, our findings indicate that reducing ChC activity increases seizure susceptibility, whereas increasing ChC activity reduces the occurrence of seizures. These findings support the hypothesis that ChCs may play a protective role against excessive excitatory activity. Enhancing ChC activity or preserving their synaptic transmission could present a potential approach to mitigate seizure activity.

### 2.4 Increasing the Activity of ChCs Attenuates Spontaneous Seizures During the Chronic Phase

Since increased activity of ChCs reduced seizure susceptibility and severity during the acute phase of KA‐induced SE, we next tested whether boosting ChC activity could also confer protection during the chronic phase. SE was initially induced via intrahippocampal injection of KA (100 ng, 0.2 µL) through a cannula, and mice that experienced SE were used for subsequent analyses during the chronic phase. Mice expressing hM3Dq in the ChCs were initially not administered CNO during the acute KA induction phase. The absence of CNO application during this phase revealed no discernible differences in the susceptibility and severity of acute seizures between the groups (Figure S6A, B, Supporting Information). This ensured that any effects resulting from manipulating ChCs during the chronic phase were not influenced by variations in acute phase severity.

**Figure 5:**
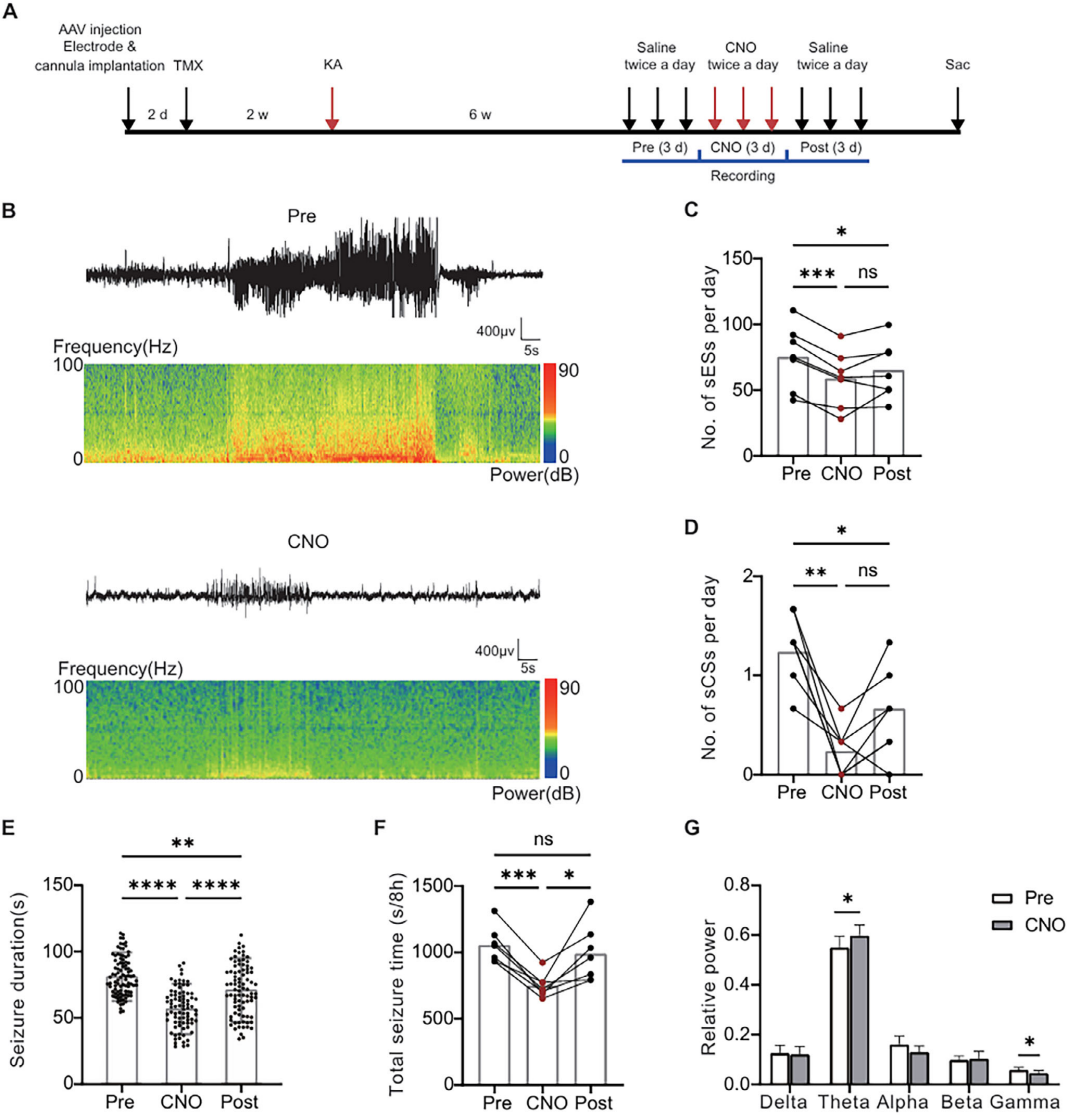
Chemogenetic activation of ChCs decreased the occurrence of spontaneous seizures during the chronic phase. A) Experimental setup illustrating the protocol to activate ChCs during the chronic phase of KA‐induced TLE. B) Representative EEG trace and corresponding power spectrum before and during CNO administration. C) The average number of the sESs per day before (Pre), during (CNO), and after (Post) CNO application in hM3Dq group (Pre versus CNO, *P* < 0.001; Pre versus Post, *P* < 0.05; CNO versus Post, *P* = 0.3591, one‐way repeated measures ANOVA, n = 7 mice). D) The average number of the sCSs per day before (Pre), during (CNO), and after (Post) CNO application in the hM3Dq expressing group (Pre versus CNO, *P* < 0.01, Pre versus Post, *P* < 0.05, CNO versus Post, *P* < 0.05, one‐way repeated measures ANOVA, n= 7 mice). Data are represented as mean with each individual value. E,F) Reduced seizure duration (E) and total time (F) of the sESs upon application of CNO (Duration: Pre versus CNO, P < 0.001; Pre versus Post, *P* < 0.05; CNO versus Post, *P* < 0.001, one‐way ANOVA, Pre n = 91, CNO n = 81, Post n = 86, from 7 mice; Total time: Pre versus CNO, *P* < 0.001; Pre versus Post, P = 0.6; CNO versus Post, *P* = 0.0365, one‐way repeated measures ANOVA, n = 7 mice). G) Relative power before (Pre) and during (CNO) the application of CNO (two‐way ANOVA, Theta: *P* = 0.041; Gamma: *P* = 0.016). ns *P* > 0.05, ^*^ *P* < 0.05, ^**^ *P* < 0.01, ^***^ *P* < 0.001, ^****^ *P* < 0.0001.

Six weeks post initial SE induction, EEG recordings were conducted over 9 consecutive days. Animals exhibiting chronic spontaneous seizures, characterized by increased EEG amplitude and frequency during the initial 3‐day recording period (pre), were further investigated (**Figure** **5**A). CNO was applied twice a day in the following 3‐day recording period (CNO), and another 3‐day recording was conducted afterward (post). In the GFP control group, both the incidence of sESs and sCSs remained relatively stable before (pre), during (CNO), and after (post) CNO administration (Figure S6C,D, Supporting Information). However, in the group injected with DIO‐hM3Dq during CNO administration, a significant reduction in both sESs and sCSs was observed (Figure 5B–D). Even three days post‐CNO application, we noticed a sustained protective effect with reduced occurrence of sESs and sCSs (Figure 5C,D). Moreover, we found that both seizure duration and total time were reduced on days when CNO was applied, with alterations in the relative power of the theta band and gamma bands during seizures following CNO application (Figure 5E–G). These findings suggest that increased activity of ChCs plays a protective role against chronic spontaneous seizures.

### 2.5 Intensified Innervation of AIS by ChC Cartridges After Status Epilepticus

**Figure 6:**
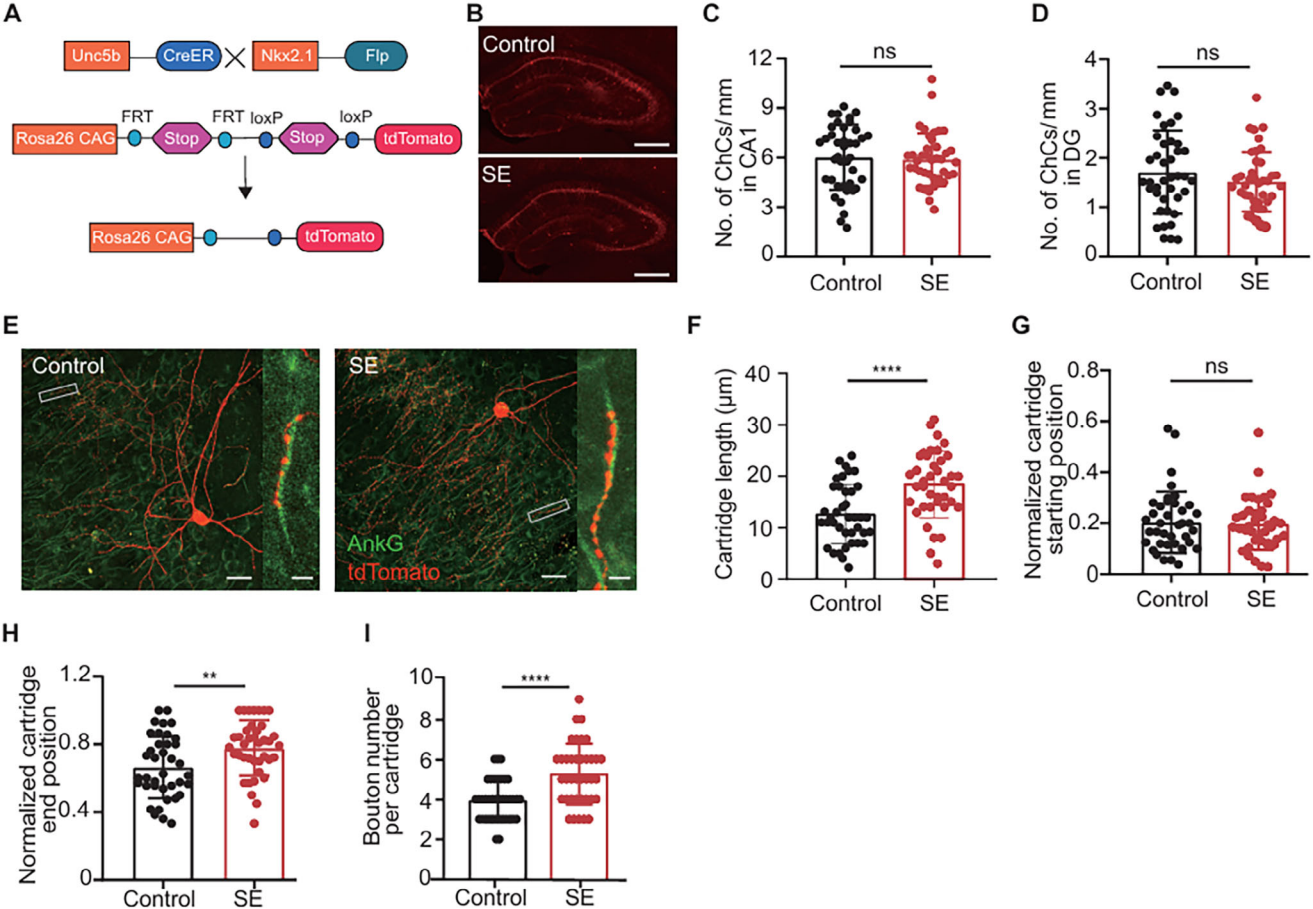
Intensified innervation of ChC cartridge to the AIS following SE. A) Illustration of the intersectional strategy employed to label ChCs: *Unc5b‐CreER* and *Nkx2.1‐Flp* driver lines were crossed with *Ai65* reporter line to achieve selective expression of tdTomato exclusively in ChCs. B) Representative images of the hippocampi showing labeled ChCs from control mice and mice subjected to the KA‐induced SE (scale bar: 500 µm). C,D) The density of ChCs in the CA1 (*P* = 0.7393) and DG regions (*P* = 0.2484) remained unchanged following SE. (Student's *t*‐test, n = 40, 8 brain slices, 5 mice). E) Confocal images displaying ChCs (tdTomato, red) and AIS (AnkG, green) in the CA1 region of control mice or mice that experienced SE. Insets illustrate enlarged AIS innervated by a cartridge from ChC (scale bar: 30 µm; insets 5 µm). F) The length of ChC cartridges was increased in the SE group (*P* < 0.0001, Student's *t*‐test, n = 39, from 3 mice). G) No significant changes were observed in the starting position of the cartridges on the AIS in SE group (*P* = 0.7168, student's *t*‐test, n = 38, from 3 mice). H) The relative end position of the cartridges moved distally in the SE group (*P* = 0.0066, student's *t*‐test, n = 38, from 3 mice). I) The number of boutons per cartridge was increased in the SE group (*P* < 0.0001, student's *t*‐test, n = 38, from 3 mice). Data are represented as mean ± SD. ^**^ *P* < 0.01, ^****^ *P* < 0.0001.

Various pathological changes have been reported in the axo‐axonic innervation of hippocampal PNs in patients with TLE. These changes encompass decreased, increased, or rearranged ChC cartridges.^[^ ^27^, ^28^, ^29^, ^30^ ^]^ The discrepancies in these findings may be attributed to the intricate origin of disease onset or secondary harm resulting from status epilepticus (SE). To explore whether SE triggers any pathological modifications in ChCs, we applied a genetic strategy to achieve a more controllable and consistent labeling of ChCs. Given that Unc5b is also expressed in capillary endothelia cells, resulting in tdTomato expression in blood vessels, we employed an intersectional approach (*Unc5b‐CreER: Nkx2.1‐Flp: Ai65*). By introducing a second driver line, *Nkx2.1‐Flp*, which has restricted expression of Flp in MGE‐derived interneurons, the signals in blood vessels were subtracted and allowed for precise and efficient labeling of ChCs (**Figure** **6A,B**; Figure S7, Supporting Information).^[^ ^34^ ^]^ Additionally, we can achieve either high‐density labeling or sparse labeling, depending on the TMX dosage employed.

To maintain the structural integrity of the hippocampus, we induced SE via i.p. injection of KA (25 mg kg^−1^). Mice that underwent SE were sacrificed 6 weeks later. First, we examined whether there were any changes in the cell density of ChCs following SE. For this purpose, we densely labeled ChCs by i.p. injections of a high dosage of TMX (100 mg kg^−1^), at postnatal day 28 (P28) and P30, respectively. Since the majority of ChC cell bodies were located within or near the stratum pyramidale (str. pyr.) layer of CA1 and the stratum granulosum (str. gr.) of the dentate gyrus (DG), we calculated the cell density of ChCs by dividing the number of ChCs by the length of the str. pyr. in CA1 and the str. gr. in DG. The cell density at the CA1 region was 6.01 ± 1.96 /mm and the one at the DG region was 1.71 ± 0.84 /mm. Following the induction of SE, the cell density at the CA1 region was 5.88 ± 1.60 /mm and the one at the DG region was 1.52 ± 0.61 /mm. No significant difference was observed between the control group and the SE group, indicating that ChCs are relatively resistant to SE‐induced damage (Figure 6B–D).

To further explore whether potential alterations happened in ChCs post‐SE, we employed sparse labeling via i.p. injection of a low dose TMX (15 mg kg^−1^). Under this condition, non‐overlapping ChCs were labeled in the CA1 region, leaving each AIS with a single cartridge labeled. This approach allowed for more accurate analysis of each cartridge (Figure 6E). Due to the low efficiency of this approach in labeling DG ChCs, our study focused on the CA1 region for subsequent analyses. We observed an increased length of ChC cartridges (Figure 6F). The elongated cartridges extended toward the distal end of the AIS, whereas the position of the most proximal bouton (marked as the starting position of a cartridge) remained unchanged (Figure 6G,H). Additionally, the average number of boutons of each cartridge increased post‐SE (Figure 6I). Interestingly, these plastic changes can be detected as early as one week post‐SE (Figure S8, Supporting Information). These consistent changes suggest that ChCs intensified their innervation to the AIS following SE, indicating their resilience to seizure‐induced damage. These results are in line with a protective role of ChCs against seizure progression during the chronic phase of epileptic seizures.

## 3 Discussion

More than two decades ago, it was proposed that the loss of ChCs might represent a key component in the development of TLE.^[^ ^26^ ^]^ Supporting this notion, the loss or reorganization of ChC cartridges has been observed in human TLE patients and animal models.^[^ ^27^, ^28^, ^29^, ^30^ ^]^ Recently, compromised ChC excitability post‐SE has been observed in the DG region in a mouse model of TLE.^[^ ^33^ ^]^ However, determining whether the changes in ChCs are a consequence of epileptic seizures or a primary process that heightens susceptibility to seizure development has proven challenging. Our findings, utilizing the newly developed genetic line that specifically targets ChCs, elucidated a protective role of ChCs in epileptic seizures. While the loss of ChCs has been observed in certain TLE patients, our observations in the KA‐induced TLE model did not show a reduction in the cell density of ChCs. This suggests that the reduction of ChCs might represent a primary event that contributes to epilepsy development, supporting the “ChC hypothesis” of seizure onset.

The loss of subpopulations of GABAergic interneurons is well documented in human TLE and animal models of TLE, which is one of the leading causes of imbalanced “excitation and inhibition”.^[^ ^28^, ^30^, ^41^, ^42^, ^43^, ^44^ ^]^ In multiple animal models of TLE, SOM^+^ and PV^+^ interneurons have been shown to be particularly vulnerable, yet with different degrees and functions.^[^ ^41^, ^42^, ^45^, ^46^ ^]^ In contrast, we observed an increase in ChC innervation to the AIS, which is consistent with the observation that ChC cartridges undergo rearrangement in the hippocampi of some TLE patients.^[^ ^30^ ^]^ The increased innervation of cartridges toward the AIS of PNs is in line with the understanding that the innervation of ChCs to the PN AIS can exhibit high plasticity, which serves to counteract disturbed network activity.^[^ ^24^ ^]^ Accordingly, our findings, demonstrating that further increasing ChC activity during the chronic phase reduced the occurrence of spontaneous seizures, support the hypothesis that ChCs participate in regulating excessive network activity. Although both PV^+^ basket cells and SOM^+^ neurons are known to exert protective roles against seizures ^[^ ^47^, ^48^, ^49^ ^]^ PV^+^ cells exhibit an immediate response of Ca^2+^ signals even before the rise of RMS (Figure S1D, Supporting Information)^[^ ^50^ ^]^, and SOM^+^ neurons exhibit a similar pattern.^[^ ^50^ ^]^ This rapid response suggests that they may play roles in different stages compared to ChCs.

As a major source of perisomatic inhibition, PV^+^ fast‐spiking interneurons constitute ≈40% of the interneuron population.^[^ ^51^ ^]^ They can be further categorized into two major subgroups: the PV^+^ basket cells, which contribute to somatic inhibition, and ChCs, which specifically innervate the AIS of PNs. The role of PV^+^ interneurons in ictogenesis is sometimes controversial, and discrepancies can be attributed to various factors such as the transmembrane chloride gradient, specific brain region being studied, the animal models being employed, and the stages of epilepsy being investigated.^[^ ^48^, ^49^, ^50^, ^52^, ^53^, ^54^, ^55^ ^]^ However, one aspect that has not been extensively discussed in these contexts is the heterogeneity of PV^+^ neurons. This is mainly due to the lack of suitable genetic tools to distinguish and manipulate specific subpopulations of PV^+^ interneurons. Recent advancements in molecular marker identification for ChCs and the subsequent development of genetic tools based on these markers have allowed for efficient labeling and manipulation of ChCs in different brain regions.^[^ ^35^, ^56^, ^57^ ^]^ By utilizing these tools, we have successfully differentiated ChCs from PV^+^ basket cells and identified their protective roles during and after the induction of SE. However, the lack of an intersectional driver line that specifically targets and manipulates PV^+^ basket cells without affecting ChCs poses a challenge in functionally dissecting the roles of these two cell types. Further investigation is needed to develop more precise tools that can selectively manipulate PV^+^ basket cells while leaving ChCs unaffected.

Recordings from dentate ChCs have revealed a decrease in their intrinsic excitability following SE, which may contribute to disease progression due to reduced inhibition from ChCs.^[^ ^33^ ^]^ Indeed, changes in intrinsic excitability serve as an important driving force for spontaneous epilepsy.^[^ ^58^, ^59^ ^]^ However, whether altered dentate ChCs are sufficient to drive ictogenesis remains unclear. The *Unc5b‐CreER* driver line labels dentate ChC with extremely low efficiency (1.71 ± 0.84 /mm) (Figure 6D), which makes it difficult to address this question directly. Our results obtained by manipulating ChCs in the CA1 region of the hippocampus indicate that hippocampal ChCs may play a central role in monitoring network activity, as evidenced by changes in seizure onset latency and the number of ictal events. The ChC hypothesis of epilepsy, proposed by Dr. DeFelipe, suggests that the somatodendritic areas of PNs receive GABAergic input from various types and a large number of interneurons, whereas the AIS primarily receives GABAergic input from one to a few ChCs. Therefore, factors that lead to a reduction of GABAergic interneurons may have a more profound impact on individuals with subtle differences in their ChC numbers or connections.^[^ ^26^ ^]^ Interestingly, the central nodes recognized for their role in controlling epilepsy, such as the amygdaloid complex, the hippocampal formation, and the piriform cortex, have a high density of ChCs.^[^ ^35^, ^60^, ^61^ ^]^ Exploring the potential correlation between the extent of ChC innervation and enrollment of the brain region in seizure control poses an intriguing question for future endeavors. The observation that manipulating ChC activity altered seizure susceptibility provides an initial step of experimental evidence supporting the ChC hypothesis of epilepsy.

In summary, this study provides direct evidence supporting the hypothesis that ChCs may possess an anti‐seizure function. They were recruited following seizure onset. Diminishing ChCs' activity during seizure induction accelerated seizure onset and heightened seizure severity, whereas activating them mitigated seizure activity. Furthermore, they were relatively resistant to seizure‐induced damage, and enhancing ChC activity reduced the occurrence of spontaneous seizures during the chronic phase, offering a potential therapeutic target to prevent the emergence of spontaneous seizures.

## 4 Experimental Section

All animal procedures were approved by the Fudan University Animal Experimentation Committee (approval number: 20210302‐145). The *Unc5b‐CreER* and *Nkx2.1‐Flp* driver lines were provided by Professor Z. Josh Huang from Duke University, School of Medicine. The *Rosa26‐frtSTOPfrt‐loxpSTOPloxp‐tdTomato* (Ai65, JAX Stock 021875) and *PV‐Cre* (JAX stock 008069) lines were from Jackson Laboratory. To genetically label ChCs*, Unc5b‐CreER* and *Nkx2.1‐Flp* driver lines were crossed with the *Ai65* intersectional reporter. For viral infection of ChCs, *the Unc5b‐CreER* line was used. All experiments were conducted using male mice aged between 8–18 weeks, and the mice were maintained under standard conditions at a room temperature of 23 ± 2 °C, a humidity of 50% ± 10%, and a 12 h light/dark cycle, with food and water available.

### Stereotaxic Surgery

The animals were anaesthetized in a chamber with 3% isoflurane and then secured to a stereotaxic frame with a continuous supply of 2% isoflurane. During the procedure, the body temperature of the mice was maintained at 37 °C using a heating pad. AAV was bilaterally injected into four sites within the CA1 region of the hippocampus (AP: ‐1.9, ML: ±1.1, DV: ‐1.25 & AP: ‐2.8, ML: ±2.4, DV: ‐1.65). The following AAV virus and their respective dosages were used in this study: AAV2/9‐hSyn‐FLEX‐EGFP (6 × 10^11^ vg mL^−1^, 200 nl each site, Taitool); AAV2/9‐hEF1α‐DIO‐mCherry‐P2A‐Tettox (6 × 10^11^ vg mL^−1^, 200 nl each site, Taitool); AAV2/9‐hSyn‐DIO‐jGCamP7s (1.3 × 10^12^ vg mL^−1^, 200 nl each site, Taitool); AAV2/9‐hSyn‐DIO‐hM3Dq‐mCherry (4 × 10^11^ vg mL^−1^, 200 nl each site, BrainCase); AAV2/9‐hSyn‐DIO‐hM4Di‐EGFP (6 × 10^12^ vg mL^−1^, 200 nl each site, Taitool). Subsequently, a cannula for KA (100 ng, 0.2 µL) injection or an optical fiber for fluorescence collection was implanted in the left CA1 region (AP: ‐1.9, ML: 1.1, DV: ‐1.15). Subdural screw EEG electrodes were positioned above the left frontal cortex (AP: +1.8, ML: +1.0) and right dorsal hippocampus (AP: ‐1.9, ML: ‐1.1) for recording purposes. Additionally, a ground electrode and a reference electrode were implanted above the right occipital lobe (AP: ‐6.0, ML: ‐2.0) and the left occipital lobe (AP: ‐6.0, ML: +2.0), respectively. All electrodes and cannulas were then secured with dental cement. The expression of viral‐encoded Cre‐dependent transgenes was induced by i.p. injection of TMX at a dosage of 100 mg kg^−1^ two days after surgery. Functional proteins were given a minimum of 2 weeks to express.

### EEG Recordings and Definitions of Seizure States

After a recovery period of 2‐3 weeks, all mice were individually housed in custom‐made soundproofed boxes for EEG recordings with simultaneous video recordings. The reference and recording electrodes were connected to multichannel cables that were tethered with a signal amplifier (Medusa, Bio‐Signal Technologies). The EEG recordings were conducted at a sample rate of 1 kHz, and a time‐locked video monitoring system ran simultaneously.

To evaluate the change in seizure threshold in the mice infected with DREADDS, CNO (5 mg kg^−1^, Sigma, USA) was administered via i.p. injection 45 min before the intrahippocampal injection of KA. This procedure was conducted following a 2 h free‐moving period, with continuous recordings carried out for an additional 48 h. To investigate the effect of altered ChC activity on spontaneous seizures during the chronic phase, CNO was administered twice daily for a consecutive 3‐day period. EEG recordings were performed for a total of 9 days, covering the time before and after the 3‐day CNO administration. This protocol was implemented 6 weeks after the initial seizure induction. The EEG data files were exported as EDF files and subsequently processed using MATLAB (Mathworks, R2020B) for further analysis.

SWDs were characterized by a rhythmic pattern that repeats at a frequency above 2 Hz, where each cycle consists of a high‐voltage spike followed by a slower wave. Seizures were identified if the RMS amplitude exceeded 3 standard deviations above the baseline, and if this elevated power activity between 10–40 Hz persisted for more than 5 s across both EEG channels.^[^ ^37^, ^62^ ^]^ SRSs were characterized by unprovoked, repetitive seizure episodes occurring in the absence of acute precipitating factors. Motor seizures were ranked by the Racine scale. Specifically, limbic motor seizures with rhythmic convulsions of the body correspond to Racine scale stage 3, while rearing with tonic‐clonic convulsions corresponds to Racine scale stage 4, and prolonged seizures culminating in SE correspond to Racine scale stage 5.^[^ ^63^ ^]^ Seizure events that meet the aforementioned electrographic features were categorized as ESs, whereas the ones that reach Racine Scale 4‐5 were classified as CSs. To further differentiate between the acute phase and chronic phase, seizure events that occur during the unprovoked chronic phase were considered spontaneous seizure events, which were categorized into spontaneous ESs (sESs) and spontaneous CSs (sCSs). To assess the duration, total time, and relative power of sESs, an 8 h period (from 10:00 AM to 6:00 PM) on the second day of the three‐day recording was examined.

The onset and offset of each focal seizure were determined by employing a change point detection algorithm in MATLAB (function “*findchangepts”*, “statistic” = “mean”, “MaxNumChanges” = 2). The power spectrum was analyzed offline using Spike2 software (Cambridge Electronic Design). The time‐frequency representation of the EEG power spectrogram was calculated by a short‐time Fast Fourier Transform (FFT) algorithm with 50% overlap using Hanning windows (sliding window length = 0.512 s, step size = 0.256 s).

### Fiber Photometry

In the simultaneous EEG and fiber photometry recordings, a fiber photometry system from Nanjing Bio‐signal was used to capture calcium events of the ChCs. Excitation light at 480 nm was delivered to the CA1 region of the hippocampus via an optical fiber, with a CMOS array collected the fluorescence signals in real‐time. The fluorescence signals were sampled at 50 Hz. Before inducing seizure via i.p. injection of KA (25 mg kg^−1^, Sigma, USA), the mice underwent 30 min of free movement. The recorded fluorescence signals were exported as MATLAB (.mat) files for subsequent analysis. The fluorescence intensity change (ΔF/F) was calculated using the formula (F−F0)/F0, where F represents the fluorescence intensity and F0 denotes the baseline fluorescence. These values were used to generate average plots and heatmaps, enabling real‐time monitoring and analysis of calcium dynamics. The processed fiber photometry data were depicted as heatmaps or displayed as fluorescence changes (ΔF/F) for visualization and analysis purposes. The EEG data were downsampled to 50 Hz to align with the time points of the fiber photometry data through interpolation (MATLAB function “interp1”, method = “pchip”). The EEG and fiber photometry data were normalized by dividing each value by its maximum value, respectively. Normalized data were aligned with corresponding time points.

For the calculation of the delay value (RMS power leads Ca^2+^ signal) when the correlation was maximal, the Ca^2+^ signal was first upsampled to 1000 Hz through interpolation (MATLAB function “interp1”, method = “pchip”), aligning it with the EEG signal's sampling rate. Subsequently, cross‐correlation analysis was performed between the power (RMS) of the EEG signal and the interpolated Ca^2+^ signal (MATLAB function “xcorr”, method = “coeff”). The results of this analysis produced a cross‐correlogram that allows us to pinpoint the delay value.

### Local Field Potential Recording

The LFP recording was performed using a custom‐made fixed‐length 32‐channel tetrode drive (8 tetrodes). Each tetrode consisted of four twisted PAC‐coated nickel‐chrome wires (12.7 µm, Sandvik, Palm Coast, USA), with each wire connected to the electrode interface board (EIB) connector (Omnetics, USA) via gold pins (Neuralynx, Bozeman, USA). Eight tetrodes (4 tetrode x 2 hemisphere) were bilaterally implanted in the dorsal CA1 (dCA1) region. To induce seizures through intrahippocampal KA delivery while simultaneously recording LFP, four tetrodes were glued around an infusion cannula to capture neuronal activity near the infusion site. The tips of the microwires extended 500 µm beyond the cannula (guide tube: ID 0.3 mm; infusion tube: ID 0.09 mm). The tetrode tips were gold electroplated (Gold Non‐Cyanide solution, Sifcoasc, Independence, USA) using the NanoZ system (Plexon Inc., Dallas, USA) to reduce impedance to 250 kΩ. Following viral injection, tetrode electrodes were implanted (AP −1.90 mm, ML ±1.10 mm, DV −1.25 mm). After surgery, mice were placed on a heating pad until recovery from anesthesia and subsequently housed individually.

Animals were allowed to recover for 2 weeks before recording. On recording day, KA (100 ng, 0.2 µL) was unilaterally infused into the hippocampus at a rate of 0.1 µL min^−1^ (Chuangrui, ZS100, China). Recordings were conducted for 2 h before and after the KA infusion using the Open‐Ephys acquisition system at a sampling rate of 30 kHz (bandpass filtering between 0.1–8850 Hz). Simultaneous video monitoring of behavior was carried out during the recordings. To mark the locations of electrode tips, an electrolytic lesion was made prior to brain dissection. This was achieved by applying a constant 10 µA current for 300 s through each selected EIB pin using a NanoZ system (Plexon, Dallas, USA).

The raw LFP data underwent down‐sampling to 1000 Hz. Specific epochs in which epileptiform discharges were not synchronized between the left and right hippocampus were selected for focal seizure analysis. The RMS of the LFP signal was calculated (MATLAB function *“envelope”*). The onset and offset of each focal seizure were determined by employing a change point detection algorithm in MATLAB (function “*findchangepts”*, “statistic” = “mean”, “MaxNumChanges” = 2). For interictal spike detection, data epochs were extracted from postictal periods of immobility or sleep, devoid of electrographic seizures. Interictal spike events were identified as spikes exceeding four standard deviations above baseline (the mean of LFP data epochs). The duration of an interictal spike was defined as the time from the initial deflection above baseline to the return to baseline. The firing rate of interictal spikes was determined by calculating the total number of detected events divided by the total duration of epochs.

### Immunohistochemistry and Image Analysis

Mice were deeply anesthetized with isoflurane inhalation (3%) and perfused transcardially with PBS followed by 4% paraformaldehyde (PFA) in 0.1 mol L^−1^ phosphate buffer (PB; pH 7.4). The brain tissue was then carefully extracted and further fixed in 4% PFA in 0.1 mol L^−1^ PB overnight at 4 °C. After fixation, the brains were cut either coronally or perpendicular to the orientation of the hippocampus into 50 µm thick sections using a Vibratome (Leica, VT1000S, Wetzlar, Germany). Immunohistochemistry was performed as previously described.^[^ ^32^, ^34^, ^64^ ^]^ Briefly, the sections were blocked with 10% newborn goat serum (NGS) and 0.3% Triton X‐100 in PBS at room temperature for 1 h, followed by incubation with primary antibodies diluted in 3% NGS and 0.3% Triton X‐100 in PBS overnight. Subsequently, sections were incubated with secondary antibodies in the same dilution buffer for 1 h at room temperature. The brain slices were then mounted onto microscope slides for image acquisition. The following primary antibodies were used: rabbit anti‐RFP (1:10000; Rockland; 600‐401‐379); mouse IgG2a anti‐AnkG (1:500; Millipore; MABN466; 3324989); chicken anti‐GFP (1:1000; Aves Labs. Inc; GFP‐1020); guinea pig anti‐PV (1:200; Synaptic Systems; 195308); mouse IgG1 anti‐SOM (1:500; GeneTex; gtx71935); and mouse IgG1 anti‐Satb1(1:200; Santa Cruz; sc‐376096). The following secondary antibodies were used: Alexa Fluor 550 goat‐anti rabbit (1:1000; Invitrogen; 84541); Alexa Fluor 488 goat‐anti mouse IgG2a secondary antibody (1:1000; Invitrogen; A‐21141); and Alexa Fluor 488 goat‐anti chicken secondary antibody (1:1000; Invitrogen; SA5‐10070).

Confocal images were acquired using a Nikon AX laser scanning microscope (Nikon, Tokyo, Japan) with 60×oil‐immersion objective (NA 1.40). To analyze the plastic changes of ChC in the CA1 region of the hippocampus, z‐stacks were acquired at a resolution of 2048 × 2048 pixels with 0.5 µm z‐intervals, ensuring complete coverage of the entire AIS and cartridge structure. The boundaries of the AIS against the soma and distal axon were determined based on positions where the normalized AnkG fluorescence intensity dropped below 0.33 of the maximum value.^[^ ^6^, ^34^ ^]^ The length of the AIS was determined by the distance between the two boundaries. The relative cartridge starting position on the AIS was calculated as the distance between the most proximal AIS bouton and the soma divided by the AIS length. Similarly, the relative cartridge end position was determined as the distance between the most distal bouton and the soma divided by the AIS length. The length of the cartridge was measured as the distance between the most proximal and distal boutons along the AIS. The number of boutons per cartridge was manually counted through the z‐stack. The ChC density was calculated by dividing the total number of ChCs located in the stratum pyramidale (str. pyr.) layer of CA1 or the stratum granulosum (str. gr.) of the dentate gyrus (DG) by the length of these respective layers.

### Statistical Analysis

Analysis was conducted in a blinded manner. Statistical analyses and data plots were performed using Prism 8.0 (GraphPad) software. Data were presented as mean ± SD. Paired or unpaired Student's t‐test was used to compare two groups. One‐way ANOVA was used to compare among three groups. Statistical significance was defined as ^*^ *P* < 0.05, ^**^ *P* < 0.01, ^***^ *P* < 0.001, ^****^ *P* < 0.0001. Values of *P* ≥ 0.05 were considered not statistically significant.

Y.L., J.T., J.W., and Q.W. contributed equally to this work. The authors thank Dr. Josh Z. Huang at Duke University for the Unc5b‐CreER and the Nkx2.1‐Flp mice, and the Allen Brain Institute for the PV‐Cre and Ai65 mice. The authors thank Dr. Miao He at Fudan University for discussion and critical reading of the manuscript. This work was supported by the National Natural Science Foundation of China (32371006, 32100795, 82071450, 32371073, and T2394531), the China Postdoctoral Science Foundation (2021M700838), the National Key R&D program of China (2024YFF1206500), and the Shanghai Pilot Program for Basic Research‐Fudan University 21TQ1400100 (25TQ002).

## Conflict of Interest

The authors declare no conflict of interest.

## Supporting information



Supporting Information

## Data Availability

The data that support the findings of this study are available on request from the corresponding author. The data are not publicly available due to privacy or ethical restrictions.
