# Supplementary material for: Hippocampal Chandelier Cells Modulate Seizure Susceptibility and Severity
Source: Adv Sci (Weinh). 2025 Oct 29;13(4):e01066. doi: 10.1002/advs.202501066 (PMC12822456; doi:10.1002/advs.202501066)
Supplement: Supplementary file 1 — Supporting Information [file ADVS-13-e01066-s001.docx]

**Supplementary Figures**

**
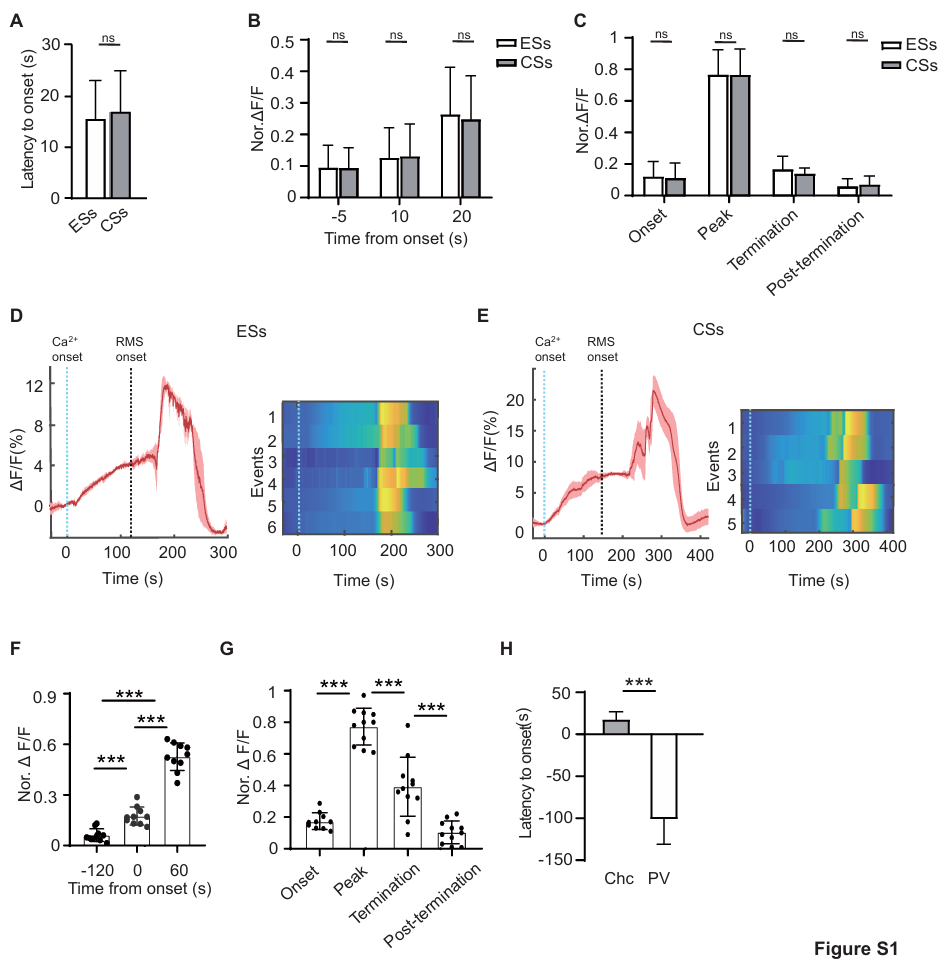
**

**Supplementary Figure 1: Ca^2+^ signal dynamics of ChCs and PV^+^ cells during ESs and CSs.** **A** Averaged latency from seizure onset to the initiation of ChC Ca^2+^ rise during ESs or CSs (Student’s *t* test, *P* = 0.47, ESs: n=6 from 3 mice; CS: n=6 from 3 mice). **B** The ChC Ca^2+^ signals significantly increase 20 seconds after the onset of seizures in both ESs and CSs. **C** The dynamics of Ca^2+^ signals are shown at four distinct time points—onset, peak, termination, and post-termination—during ESs or CSs. **D** Representative trace of average Ca^2+^ signal of PV^+^ neurons during ESs. Blue and black dash lines indicate the onset of the rise of Ca2+ signal and RMS signal respectively. Heatmap on the right panel showed Ca2+ signals of each ES incident (n = 6, from 3 mice). **E** Representative trace of average Ca^2+^ signal of PV^+^ neurons during CSs. Blue and black dash lines indicate the onset of the rise of Ca^2+^ signal and RMS signal respectively. Heatmap on the right panel showed Ca^2+^ signals of each ES incident (n = 5, from 3 mice). **F** Normalized ΔF/F of Ca^2+^ signals -120 sec before onset, at the onset, and 60 sec after onset of the seizure events (One-way ANOVA, -120 sec vs. 0 sec, *P* < 0.001; 0 sec vs. 60 sec, *P* < 0.001; -120 sec vs. 60 sec, *P* < 0.001; n = 11, from 3 mice). **G** Changes of Ca^2+^ signals of PV^+^ neurons at seizure onset, peak, termination and post-termination (One-way ANOVA, onset vs. peak, *P* < 0.001; peak vs. termination, *P* < 0.001; termination vs. post-termination, *P* < 0.001; n = 11, from 3 mice) **H** Averaged latency from seizure onset to the initiation of Ca^2+^ rise of ChCs and PV^+^ neurons (Student’s t test, *P* < 0.001 ChCs: n = 12, from 3 mice; PV^+^ neurons: n = 11, from 3 mice). Data are represented as mean ± SD. ns *P* > 0.05, **P* < 0.05, ** *P* < 0.01, *** *P* < 0.001.


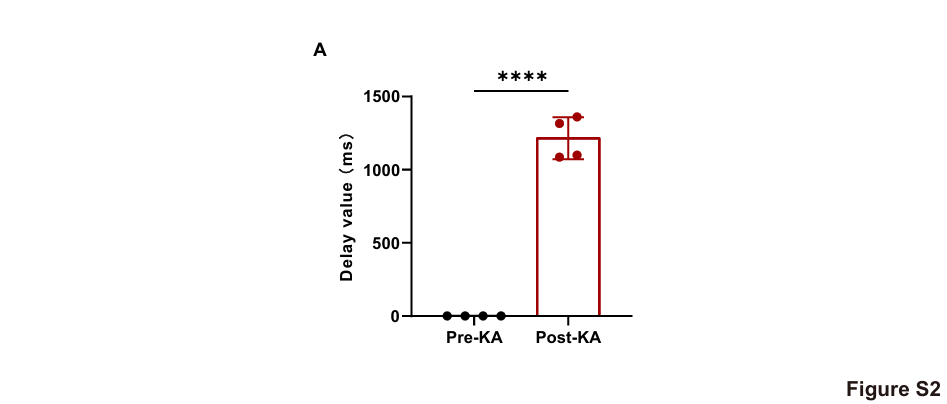


**Supplementary Figure 2: Cross-correlation analysis revealed delay value (RMS power leads Ca^2+^ signal) at which the correlation is maximal before and after KA administration.** A notable difference in delay values was observed before and after KA administration (Student’s *t* test, *P* < 0.0001, n = 4). *****P* < 0.0001


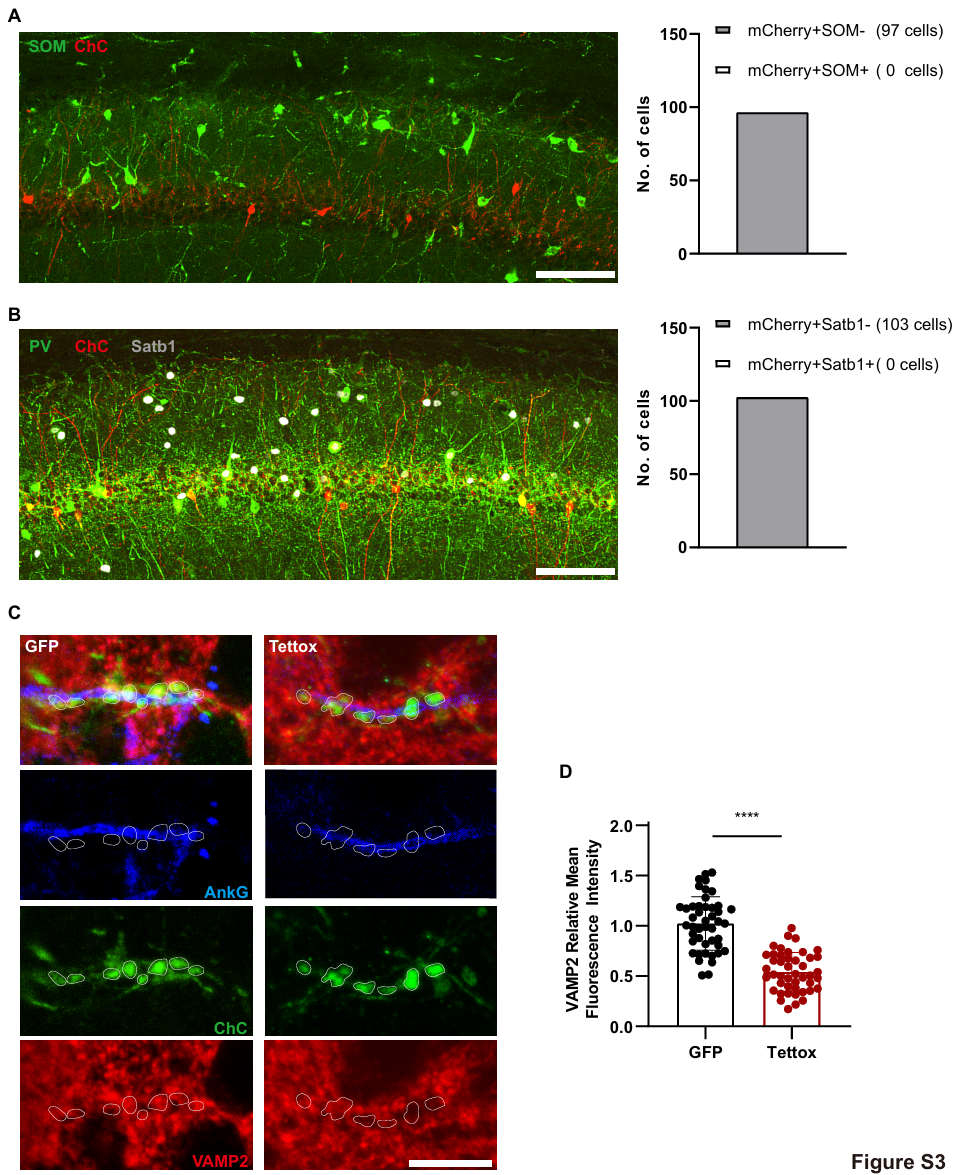


**Supplementary Figure 3: Selective targeting of ChCs in *Unc5b-CreER* mice with AAV-DIO-Tettox effectively cleaved VAMP2 in ChC boutons. A** Representative image (left) of CA1 region of *Unc5b-CreER* mice infected with AAV-DIO-Tettox (red) immunostained with anti-SOM antibody (green), along with the quantification of mCherry^+^ SOM^-^ cells and mCherry^+^ SOM^+^ cells (right, 97 cells from 3 mice). Scale bar: 100μm. **B** Representative image (left) of CA1 region of *Unc5b-CreER* mice infected with AAV-DIO-Tettox (red) immunostained with antibodies against PV (green) and Satb1(grey), along with the quantification of mCherry^+^/Satb1^-^ cells (ChCs) and mCherry^+^ /PV^+^Satb1^+^ cells (PV^+^ basket cells) (right, 103 cells from 3 mice). mCherry^-^PV^+^Satb1^-^ cells may represent unlabeled ChCs. Scale bar: 100μm. **C** Representative images of ChC boutons infected with AAV-DIO-GFP or AAV-DIO-Tettex-mCherry (pseudo-colored in green) immunostained with anti-VAMP2 antibody (red). Scale bar: 10μm. **D** Normalized mean fluorescence intensity of VAMP2 signal within the ChC boutons in indicated experimental groups (GFP: n = 45 boutons, from 3 mice; Tettox: n = 45 boutons, from 3 mice; *P* < 0.0001. Student’s *t* test). Data are represented as mean ± SD. **** *P* < 0.0001.


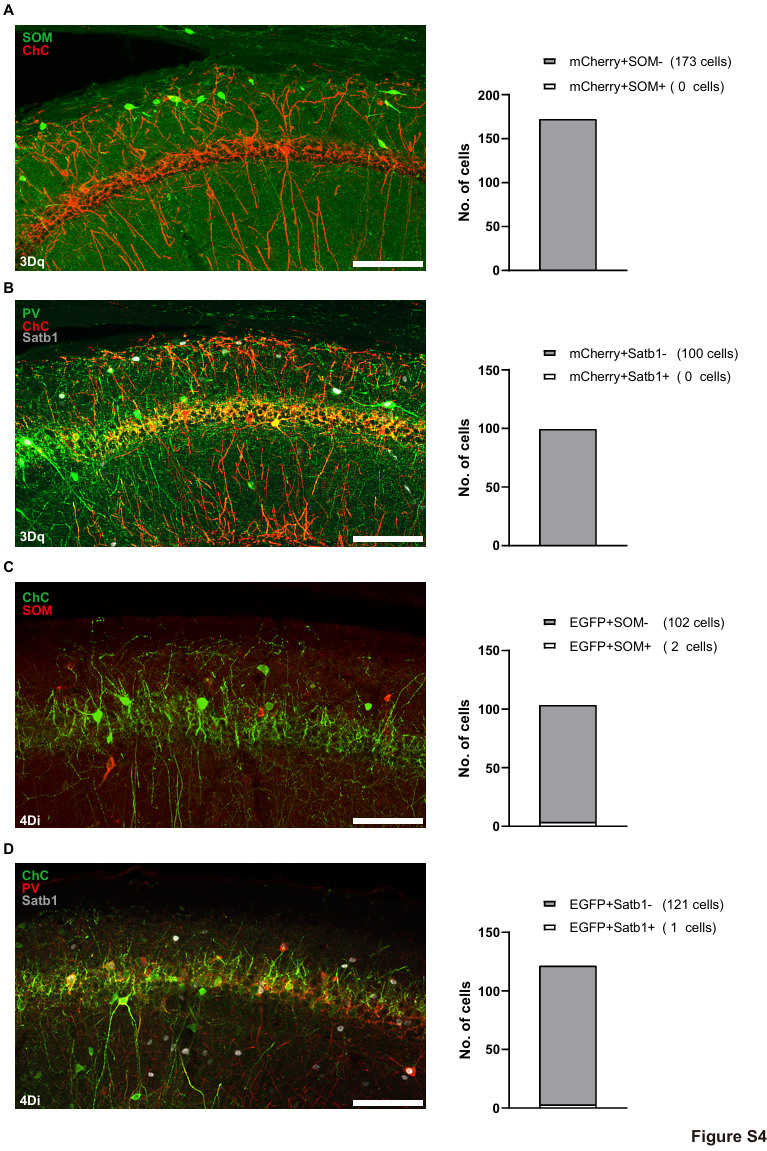


**Supplementary Figure 4: Selective targeting of CA1 ChCs with AAV-DIO-hM3Dq-mCherry or AAV-DIO-hM4Di-EGFP in *Unc5b-CreER* mice. A** Representative image (left) of CA1 region of *Unc5b-CreER* mice infected with AAV-DIO-hM3Dq-mCherry (red) immunostained with anti-SOM antibody (green), along with the quantification of mCherry^+^ SOM^-^ cells and mCherry^+^ SOM^+^ cells (right, 173 cells from 3 mice). Scale bar:100μm. **B** Representative image (left) of CA1 region of *Unc5b-CreER* mice infected with AAV-DIO-hM3Dq-mCherry (red) immunostained with antibodies against PV (green) and Satb1(grey), along with the quantification of mCherry^+^Satb1^-^ cells and mCherry^+^/PV^+^Satb1^+^ cells (PV^+^ basket cells) (right, 100 cells from 3 mice). mCherry^-^PV^+^Satb1^-^ cells may represent unlabeled ChCs. Scale bar:100 μm. **C** Representative image (left) of CA1 region of *Unc5b-CreER* mice infected with AAV-DIO-hM4Di-EGFP (green) immunostained with anti-SOM antibody (red), along with the quantification of EGFP^+^ SOM^-^ cells and EGFP^+^ SOM^+^ cells (right, 104 cells from 3 mice). Scale bar:100μm. **D** Representative image (left) of CA1 region of *Unc5b-CreER* mice infected with AAV-DIO-hM4Di-EGFP (green) immunostained with antibodies against PV (red) and Satb1(grey), along with the quantification of EGFP^+^/Satb1^-^ cells and EGFP^+^ /PV^+^Satb1^+^ cells (PV^+^ basket cells) (right, 122 cells from 3 mice). mCherry^-^PV^+^Satb1^-^ cells may represent unlabeled ChCs. Scale bar: 100μm.


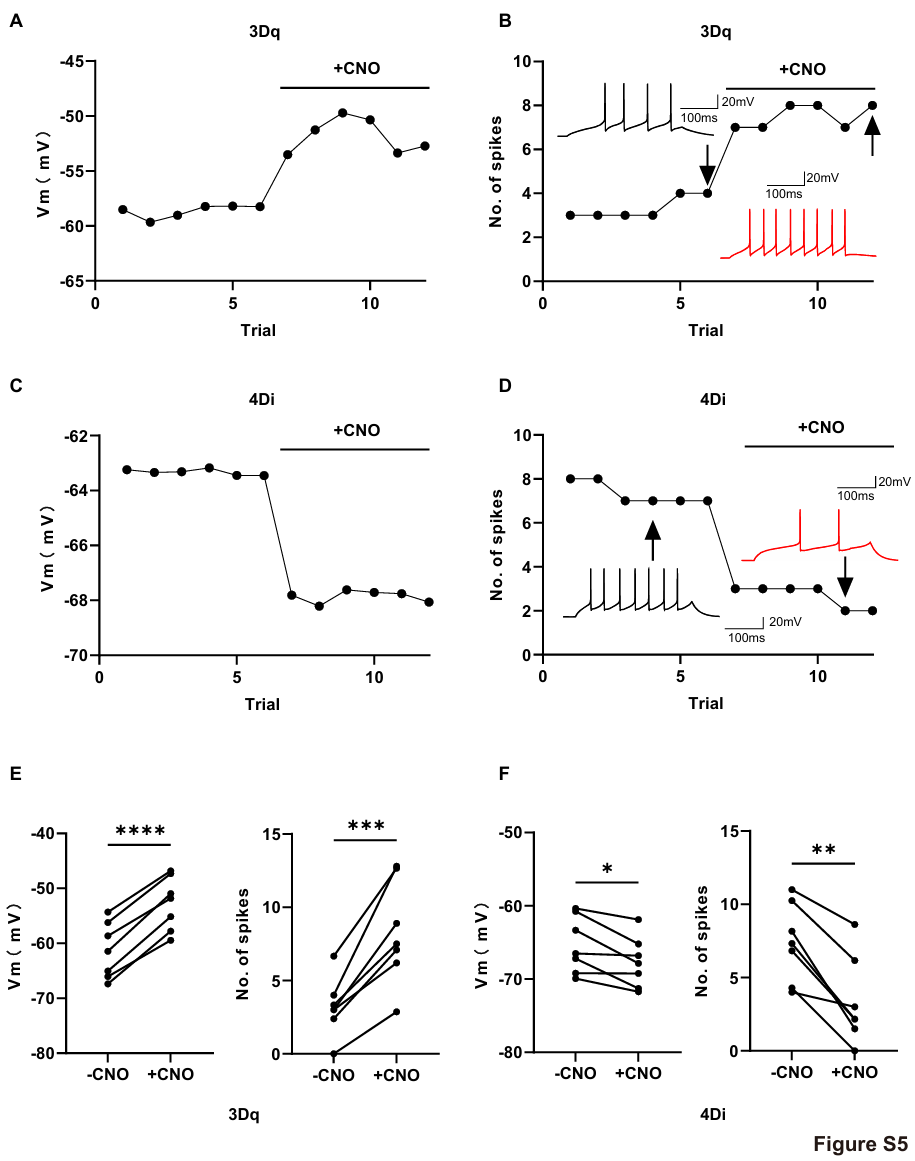


**Supplementary Figure 5: Verification of the efficacy of chemogenetic manipulation of ChCs.** Whole-cell patch-clamp recordings were performed on virally infected ChCs in acute brain slices. A constant depolarizing current (20–100 pA; adjusted individually for each cell) was injected for 300 ms at 20-second intervals to evoke approximately five action potentials per trial. After achieving a stable baseline with at least six consecutive trials, CNO was applied through the perfusing ACSF. The resting membrane potential and the number of evoked spikes were quantified for each current injection. **A-B** Representative traces illustrating the membrane potential (**A**) and firing rate (**B**) of ChCs expressing hM3Dq before and after CNO application, with 30 pA, 300 ms current injection each time. **C-D** Representative traces illustrating the membrane potential (**C**) and firing rate (**D**) of ChCs expressing hM4Di before and after CNO application, with 80 pA, 300 ms current injection each time. **E** Depolarized membrane potential (left) and increased firing rate (right) of ChCs expressing hM3Dq after CNO application (paired *t*-test; Vm: *P* < 0.0001; firing rate: P < 0.001; n = 7 cells, from 3 mice). **F** Hyperpolarized membrane potential (left) and decreased firing rate (right) of ChCs expressing hM4Di after CNO application (paired *t*-test; Vm: *P* = 0.0178; firing rate: P < 0.01; n = 7 cells, from 3 mice). * *P* < 0.05, ** *P* < 0.01, *** *P* < 0.001, **** *P* < 0.0001.


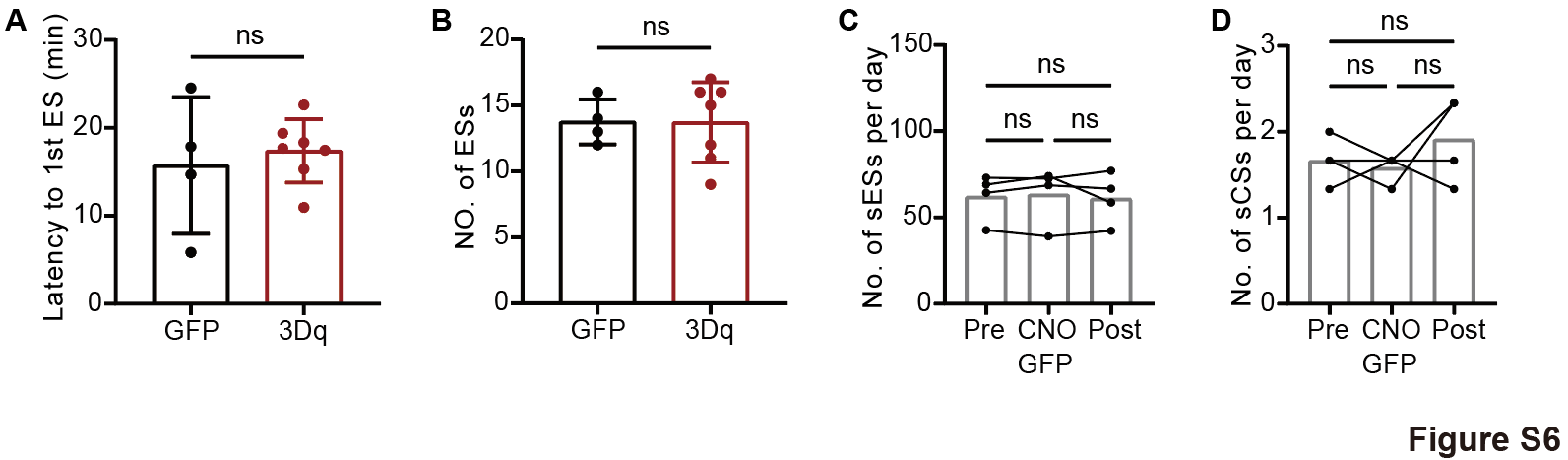


**Supplementary Figure 6: No difference was detected in the GFP and hM3Dq groups without CNO administration**. **A-B** Statistics showing the latency to the first ES (**A**, *P* = 0.634) and the frequency of ESs (**B**, *P* = 0.983) within 2 hours following KA administration in the GFP and hM3Dq groups (GFP: n = 4; hM3Dq: n = 7, student’s *t*-test). **C** The average number of the sESs per day before (Pre), during (CNO), and after (Post) CNO application in the GFP control group (Pre vs. CNO, *P* = 0.9992; Pre vs. Post, *P* = 0.9995; CNO vs. Post, *P* = 0.9948, n = 4 mice;). **D** The average number of the sCSs per day before (Pre), during (CNO), and after (Post) CNO application in the GFP control group (Pre vs. CNO, *P* = 0.8666; Pre vs. Post, *P* = 0.7327; CNO vs. Post, *P* = 0.5788, one-way repeated measures ANOVA, n = 4 mice). ns *P* > 0.05.


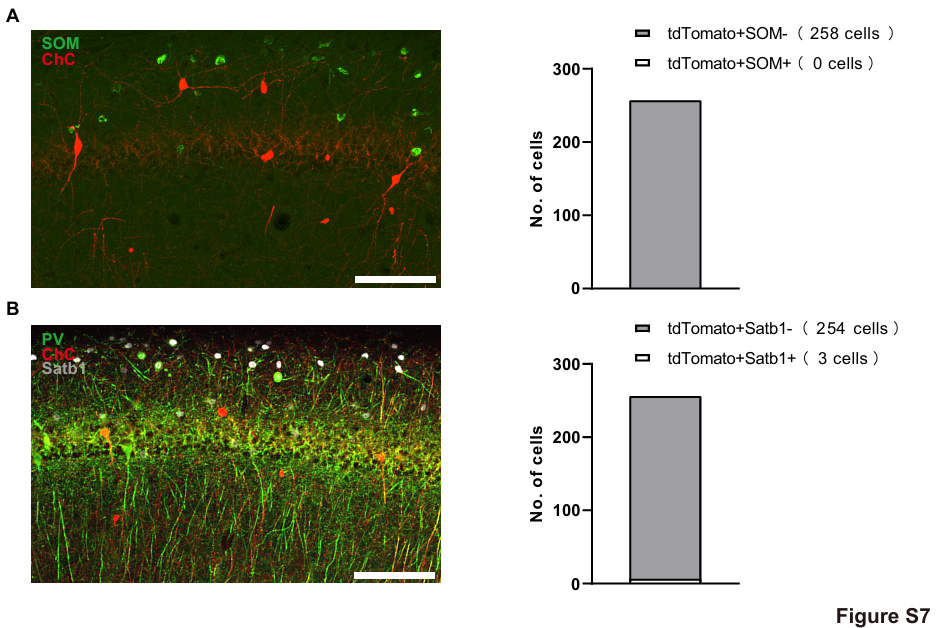


**Supplementary Figure 7: The intersectional genetic approach of *Unc5b-CreER:Nkx2.1-Flp:Ai65* selectively labeled ChCs. A** Representative image (left) of labeled cells (red) in CA1 region of *Unc5b-CreER:Nkx2.1-Flp:Ai65* mice immunostained with anti-SOM antibody (green), along with the quantification of tdTomato^+^ SOM^-^ cells and tdTomato^+^ SOM^+^ cells (right, 258 cells from 3 mice). Scale bar: 100μm. **B** Representative image (left) of labeled cells (red) in CA1 region of *Unc5b-CreER:Nkx2.1-Flp:Ai65* mice immunostained with antibodies against PV (green) and Satb1(grey), along with the quantification of tdTomato^+^Satb1^-^ cells and tdTomato^+^/PV^+^Satb1^+^ cells (PV^+^ basket cells) (right, 257 cells from 3 mice). mCherry^-^PV^+^Satb1^-^ cells may represent unlabeled ChCs. Scale bar: 100μm.


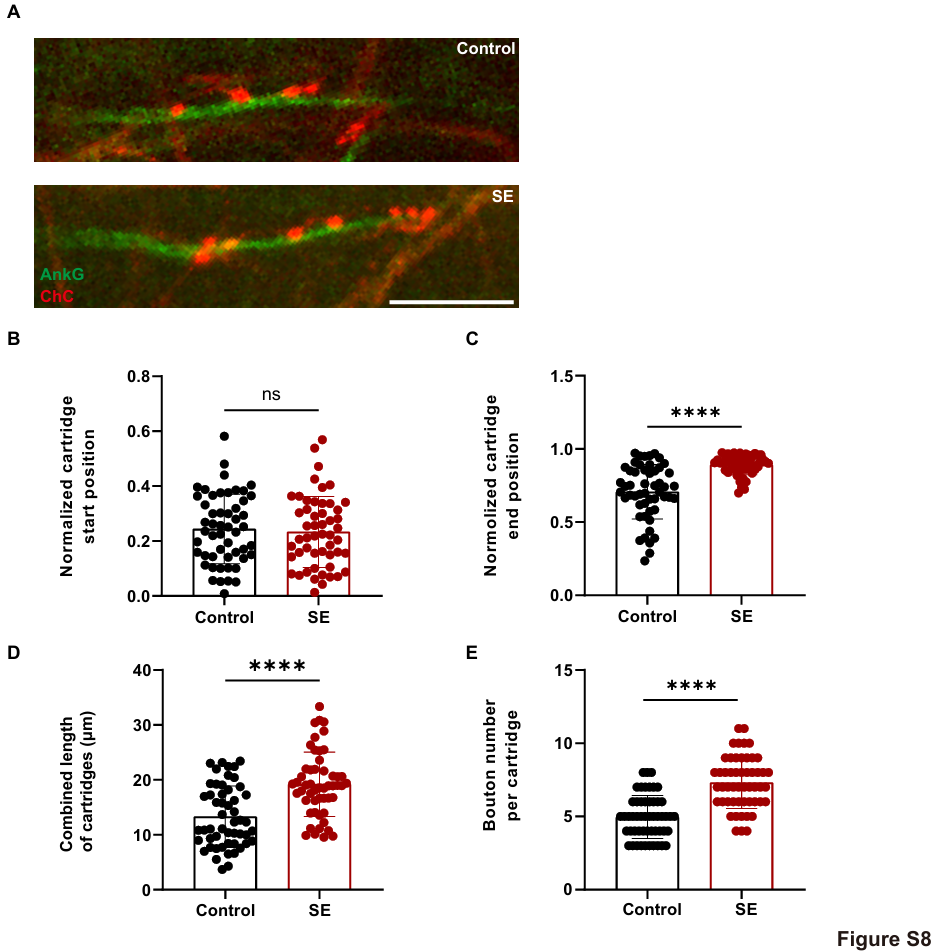


**Supplementary Figure 8: Intensified innervation of ChC cartridges to the AIS one week after SE. A** Representative images displaying ChCs cartridge (tdTomato, red) and AIS (AnkG, green) in the CA1 region of control mice and mice one week after experiencing SE. (scale bar: 10 µm). **B** No significant changes were observed in the starting position of the cartridges on the AIS in SE group (*P* =0.6667, student’s *t*-test, control: n = 52; SE: n = 52, from 3 mice). **C** The relative end position of the cartridges moved distally in the SE group (Student’s *t* test, *P* < 0.0001, control: n = 52; SE: n = 52, from 3 mice). **D** The length of ChC cartridges was increased in the SE group (Student’s *t* test, *P* < 0.0001, control: n = 52; SE: n = 52, from 3 mice). **E** The number of boutons per cartridge was increased in the SE group (Student’s *t* test, *P* < 0.0001, control: n = 52; SE: n = 52, from 3 mice). Data are represented as mean ± SD. ns *P* > 0.05, **** *P* < 0.0001.
